# Exploration of Chronic Kidney Disease Prevalence Estimates Using New Measures of Kidney Function in the Health Survey for England

**DOI:** 10.1371/journal.pone.0118676

**Published:** 2015-02-20

**Authors:** Simon D. S. Fraser, Grant Aitken, Maarten W. Taal, Jennifer S. Mindell, Graham Moon, Julie Day, Donal O’Donoghue, Paul J. Roderick

**Affiliations:** 1 Academic Unit of Primary Care and Population Sciences, Faculty of Medicine, University of Southampton, Southampton, SO16 6YD, United Kingdom; 2 Geography & Environment, Faculty of Social and Human Sciences, University of Southampton, Southampton, SO171BJ, United Kingdom; 3 Division of Medical Sciences and Graduate Entry Medicine, University of Nottingham at Derby, Derby, DE22 3DT, United Kingdom; 4 Research Department of Epidemiology and Public Health, UCL (University College London), London, WC1E 6BT, United Kingdom; 5 Department of Clinical Biochemistry, Royal Victoria Infirmary, Newcastle upon Tyne Hospitals NHS Foundation Trust, Newcastle upon Tyne, NE1 4LP, United Kingdom; 6 Renal Unit, Salford Royal NHS Foundation Trust, Salford, M6 8HD, United Kingdom; School of Public Health of University of São Paulo, BRAZIL

## Abstract

**Background:**

Chronic kidney disease (CKD) diagnosis relies on glomerular filtration rate (eGFR) estimation, traditionally using the creatinine-based Modification of Diet in Renal Disease (MDRD) equation. The Chronic Kidney Disease Epidemiology Collaboration (CKDEPI) equation performs better in estimating eGFR and predicting mortality and CKD progression risk. Cystatin C is an alternative glomerular filtration marker less influenced by muscle mass. CKD risk stratification is improved by combining creatinine eGFR with cystatin C and urinary albumin to creatinine ratio (uACR). We aimed to identify the impact of introducing CKDEPI and cystatin C on the estimated prevalence and risk stratification of CKD in England and to describe prevalence and associations of cystatin C.

**Methods and Findings:**

Cross sectional study of 5799 people in the nationally representative 2009 and 2010 Health Surveys for England. Primary outcome measures: prevalence of MDRD, CKDEPI and cystatin C-defined eGFR<60ml/min/1.73m^2^; prevalence of CKD biomarker combinations (creatinine, cystatin C, uACR). Using CKDEPI instead of MDRD reduced the prevalence of eGFR<60ml/min/1.73m^2^ from 6.0% (95% CI 5.4–6.6%) to 5.2% (4.7–5.8%) equivalent to around 340,000 fewer individuals in England. Those reclassified as not having CKD evidenced a lower risk profile. Prevalence of cystatin C eGFR<60ml/min/1.73m^2^ was 7.7% and independently associated with age, lack of qualifications, being an ex-smoker, BMI, hypertension, and albuminuria. Measuring cystatin C in the 3.9% people with CKDEPI-defined eGFR<60ml/min/1.73m^2^ without albuminuria (CKD Category G3a A1) reclassified about a third into a lower risk group with one of three biomarkers and two thirds into a group with two of three. Measuring cystatin C in the 6.7% people with CKDEPI eGFR >60ml/min/1.73m^2^ with albuminuria (CKD Category G1-2) reclassified almost a tenth into a higher risk group.

**Limitations:**

Cross sectional study, single eGFR measure, no measured (‘true’) GFR.

**Conclusions:**

Introducing the CKDEPI equation and targeted cystatin C measurement reduces estimated CKD prevalence and improves risk stratification.

## Introduction

The introduction in 2002 of a definition and classification system for chronic kidney disease (CKD) based on estimated glomerular filtration rate (eGFR) created a need for accurate methods to estimate GFR; subsequently the Modification of Diet in Renal Disease (MDRD) estimating equation was adopted worldwide.[1–4] In the UK, the National Service Framework for Renal Services 2004/05 led to national reporting of eGFR by clinical biochemistry laboratories from 2006 [5]; the General Practice pay for performance Quality Outcomes Framework (QOF) included targets for CKD management from 2006/07 [6]; and the NHS Vascular Checks Programme, introduced in 2009, includes screening for CKD (stage 3–5) in people aged 35–74 with newly identified type 2 diabetes or hypertension.[7] Nevertheless, concern has been expressed that these methods of CKD classification and identification have resulted in over-diagnosis and unnecessary disease-labelling and treatment, especially in the elderly.[8]

The more recently developed Chronic Kidney Disease Epidemiology Collaboration (CKDEPI) serum creatinine (Scr) equation has been shown to improve accuracy of estimation of eGFR and of prediction of mortality risk and risk of progression to end stage kidney disease (ESKD) over the MDRD equation.[9,10] As a result, the 2012 Kidney Disease Improving Global Outcomes (KDIGO) recommendations advocate the routine use of the CKDEPI equation for reporting of eGFR as do the recently revised CKD guidelines from the UK National Institute of Health and Care Excellence (NICE).[11,12]In a large retrospective study of routine creatinine requests, O’Callaghan et al showed that routine use of CKDEPI Scr equation in place of MDRD in a UK clinical biochemistry laboratory would result in a lower overall prevalence of CKD, but an increase in higher risk CKD stage 3–5 among older people.[13] However, the study was not able to derive population prevalence of CKD for each equation nor to assess proteinuria, an important independent risk factor.[14,15]

Scr levels are affected by factors such as diet and muscle mass, and, despite the use of weighting for age, gender and race in the eGFR estimating equations, this can result in misclassification of patients.[16,17] Serum cystatin C, another marker of glomerular filtration, is less influenced by these factors, so is an alternative for estimating GFR.[18,19] There is evidence of its improved diagnostic accuracy for impaired renal function compared with Scr and as an independent predictor of mortality risk in older people.[20] Targeted cystatin C testing has been recommended in the revised NICE guidelines where CKD diagnosis is uncertain.[12] Nationally representative serum cystatin C levels have been determined in the US by analysis of samples from the third National Health and Nutrition Examination Survey (NHANES), but population distributions of cystatin C and estimates of cystatin C-defined CKD in other developed countries are limited.[21] Equations combining Scr and cystatin C eGFR have shown improved accuracy in CKD diagnosis, and Peralta has shown improvement in risk stratification by combining Scr eGFR with cystatin C and albuminuria, demonstrating poorer outcomes in those with combinations of abnormal biomarkers.[22–24]

Using nationally representative population samples (the 2009 and 2010 Health Surveys for England (HSE)), the primary aim of this study was to examine the effect of introducing two new measures of kidney function (CKDEPI and targeted use of cystatin C) on the estimated prevalence of eGFR<60ml/min/1.73m^2^ in the general population in England. Secondary aims were to describe the distribution and associations of cystatin C, and to explore the impact of adding targeted cystatin C measurement to SCr eGFR and urine albumin to creatinine ratio (uACR) measurements on the diagnosis and risk assessment of CKD.

## Methods

Full details of the conduct of the HSE, measurement of non-CKD variables and response rates are given in the HSE 2009 and 2010 reports.[25,26] In brief, a nationally representative sample was selected each year using a stratified, two-stage sample of private addresses. Participants completed an interview questionnaire; most consented to a nurse visit. Approval was obtained from the Oxford B Research Ethics Committee for both surveys (HSE 2009 ref 08/H0605/103, HSE 2010 ref 09/H0605/73).

A random urine sample was obtained from 88% of men and 86% of women aged 16 and over who had a nurse visit, and a non-fasting blood sample from 77% of men and 73% of women. 5799 had valid serum creatinine, 5802 had valid cystatin C (measured on stored blood), 7592 had uACR results, and 5318 had valid results for all three markers.

Demographic factors included age, sex and ethnicity (self-reported). Ethnicity was grouped as White, South Asian, Black and Other. Socio-economic factors selected were: i) occupation using National Statistics Socioeconomic Classification (NSSEC, divided into three categories: high (managerial and professional occupations), middle (intermediate occupations), and low (routine and manual occupations)); ii) education qualifications grouped as: degree (NVQ4/NVQ5/Degree or equivalent), below degree, and no qualification; and iii) area-level deprivation (using Index of Multiple Deprivation 2007 (IMD) national quintiles: 1 least deprived (IMD 0.37–8.32), 2 (8.32–13.75), 3 (13.75–21.22), 4 (21.22–34.42), 5 most deprived (34.42–85.46)).[27]

Behavioural factors included self-reported smoking, and measured body mass index (BMI, defined as normal (<25kg/m^2^), overweight (25–29.9kg/m^2^), and obese (≥30kg/m^2^))[27] and waist circumference (classified as: <94cm, 94–102cm (high), and >102cm (very high) for men, and <80cm, 80–88cm (high) and >88cm (very high) for women). For South Asians, the high waist circumference threshold was 90cm for men and 80cm for women.[28]

Clinical factors included hypertension, diabetes and cholesterol level (total and high density lipoprotein (HDL)). Hypertension was defined as self-reported doctor diagnosis (pre-existing diagnosis), survey-defined (identified as having high blood pressure (BP systolic ≥140mmHg and/or diastolic ≥90mmHg and/or taking medication for hypertension) at the survey examination), and total (doctor + survey diagnosed). Diabetes was defined as self-reported doctor diagnosis (pre-existing diagnosis), survey-defined HBA1c ≥6.5% at nurse visit, and total (doctor + survey diagnosed). Serum total and HDL cholesterol levels were treated as continuous variables for regression analyses.

### Kidney function

Serum creatinine was assayed using an IDMS traceable enzymatic assay in a single laboratory (Clinical Biochemistry Department at the Royal Victoria Infirmary (RVI), Newcastle-upon-Tyne). Standard formulae were used to calculate MDRD and CKDEPI equations.[2,9] Albuminuria was measured on a single random urine sample and any albuminuria was defined as uACR >2.5mg/mmol in men and >3.5 in women (an artefact in the way normal uACR was recorded in the survey precluded use of the gender-independent KDIGO definition of >3mg/mmol).[1,3] Details of laboratory analysis, internal quality control, and external quality assurance are provided in HSE documentation.[25,26] The Roche Tina-quant immuno-turbidimetric assay was used to measure cystatin C.[29] The Grubb equation was used to derive eGFR from cystatin C values.[30] As a sensitivity analysis we also derived a threshold cystatin C level to compare with CKD defined by Grubb eGFR. Individuals in age grouping 16–34 with no hypertension, no diabetes and no albuminuria and CKDEPI eGFR≥60ml/min/1.73m^2^ were selected to determine a cut off value for increased cystatin C levels. This method is similar to that previously used in analysis of NHANES data.[21] The KDIGO classification of CKD was used to categorise eGFR.[1] Where numbers were small, individuals were categorised into four eGFR groups to allow for comparison between cystatin C and SCr-based eGFR categories (≥90ml/min/1.73m^2^; 60–89ml/min/1.73m^2^; 45–59ml/min/1.73m^2^ (G3a); and <45ml/min/1.73m^2^). Those with eGFR>60ml/min/1.73m^2^ were combined for the purposes of analysis.

### Statistical analysis

Descriptive statistics were used to compare the socio-demographic and clinical characteristics of the study population, including the distribution of cystatin C values. Overall and age-sex adjusted prevalence of eGFR<60ml/min/1.73m^2^ was compared by eGFR calculation method (MDRD vs. CKDEPI). Estimated (age-sex adjusted) numbers of people with CKD in England was derived for each method using 2011 Census data.[31]

The distribution of biomarkers in the study population was summarised using a method similar to that used by Peralta et al.[24] We identified the change in future potential risk (using Peralta’s categories of biomarker combinations) by the count of combinations of three biomarkers (CKDEPI Scr eGFR <60ml/min/1.73m^2^, uACR≥ 3mg/mmol and cystatin C eGFR <60ml/min/1.73m^2^) and summarised the effect on stratification of people in the following groups:

- People with one abnormal biomarker (creatinine based eGFR <60ml/min/1.73m^2^ but without albuminuria **or** creatinine based eGFR >60ml/min/1.73m^2^ with albuminuria) reclassified as one or two biomarkers by having cystatin C eGFR greater or less than 60ml/min/1.73m^2^:
- People with two biomarkers (creatinine-based eGFR <60ml/min/1.73m^2^ **and** albuminuria) reclassified as two or three biomarkers by having cystatin C eGFR greater or less than 60ml/min/1.73m^2^:

Univariate, age-sex adjusted, and multivariable logistic regression models were used to examine the associations between cystatin C eGFR (Grubb) and demographic, socio-economic, lifestyle and clinical factors. Age/sex, age/socioeconomic status, and age/diabetes interactions were tested.

Overall CKD prevalence estimation accounted for weighting within gender to allow for gender differences in response. Odds ratios are presented with 95% confidence intervals (CIs) and p values <0.05 are considered statistically significant unless otherwise stated. All analyses were performed using IBM SPSS Statistics version 19.

## Results

The household response rates of the 2009 and 2010 HSE were 68% and 66% respectively.[24,25] The study population consisted of 5799 individuals (5786 weighted population) with both serum creatinine and cystatin C results. 3186/5799 (55%) were women and 2613/5799 (45%) were men; they were predominantly white; and the median age was 50 (interquartile range 38–64) (Table 1). These characteristics were similar for 7952 people with a valid uACR and 5260 with all three biomarkers.

**Table 1:** Sociodemographic and clinical characteristics of the weighted study sample.

Variable	Category	People with valid serum creatinine and cystatin c value	
		Number	(%) in category
All	Aged 16+	5799	(100%)
**Age**	Age 16–34	1756	(30.3%)
	Age 34–54	2037	(35.2%)
	Age 55–64	856	(14.8%)
	Age 65–74	615	(10.6%)
	Age 75+	522	(9.0%)
**Ethnicity**	White	5244	(90.7%)
	South Asian	243	(4.2%)
	Black	154	(2.7%)
	Other	139	(2.4%)
**Sex**	Male	2823	(48.8%)
	Female	2963	(51.2%)
**Occupation (NS-SEC)**	High	1894	(33.1%)
	Middle	1203	(21.0%)
	Low	2619	(45.8%)
**Qualification**	Degree	1295	(22.4%)
	Below degree	3296	(57.0%)
	None	1197	(20.6%)
**IMD quintile** (1 = least deprived, 5 = most deprived)	1. (IMD 0.37–8.32)	1197	(20.7%)
	2. (IMD 8.32–13.75)	1204	(20.8%)
	3. (IMD 13.75–21.22)	1228	(21.2%)
	4. (IMD 21.22–34.42)	1105	(19.1%)
	5. (IMD 34.42–85.46)	1051	(18.2%)
**Smoking**	Never	3126	(54.2%)
	Ex	1429	(24.8%)
	Current	1210	(21.0%)
**Body mass index (BMI)** (kg/m^2^)	Mean ±SD	27.14	±5.06
**BMI categories**	Normal	1956	(36.8%)
	Overweight	2047	(38.5%)
	Obese	1314	(24.7%)
**Waist circumference** (cm)	Mean ±SD	92.92	±14.01
**Waist circumference categories**	Low	2120	(37.1%)
	High	1347	(23.6%)
	Very High	2242	(39.3%)
**Total cholesterol** (mmol/L)	Mean ±SD	5.30	±1.44
**Total cholesterol categories**	< 5mmol/L	2675	(46.2%)
	≥ 5mmol/L	3110	(53.8%)
**HDL cholesterol** (mmol/L)	Mean ±SD	1.48	±0.43
**HDL cholesterol categories**	< 1.2mmol/l	1301	(22.5%)
	≥ 1.2mmol	4485	(77.5%)
**Albuminuria**	None	4837	(92.0%)
	Micro	399	(7.6%)
	Macro	22	(0.4%)
**Diabetes**	No diabetes	5370	(92.6%)
	Doctor diagnosed^a^	305	(5.3%)
	Survey defined ^b^	316	(5.5%)
	Total ^c^	429	(7.4%)
**Hypertension**	No hypertension	3800	(65.5%)
	Doctor diagnosed ^a^	1387	(23.9%)
	Survey defined ^d^	1542	(26.6%)
	Total ^c^	1980	(34.1%)

Figures are number (%) unless stated otherwise

^a^ Self-reported doctor diagnosis

^b^ HBA_1c_ ≥6.5%

^c^Doctor or survey diagnosed

^d^ Identified as high blood pressure (BP systolic ≥140mmHg and/or diastolic ≥90mmHg and/or taking medication for hypertension)

NS-SEC: National Statistics Socioeconomic Classification

IMD: Index of Multiple Deprivation

BMI: Body Mass Index

HDL: High Density Lipoprotein.

### Effect on estimated prevalence of introducing new measures of kidney function

**Fig 1:**
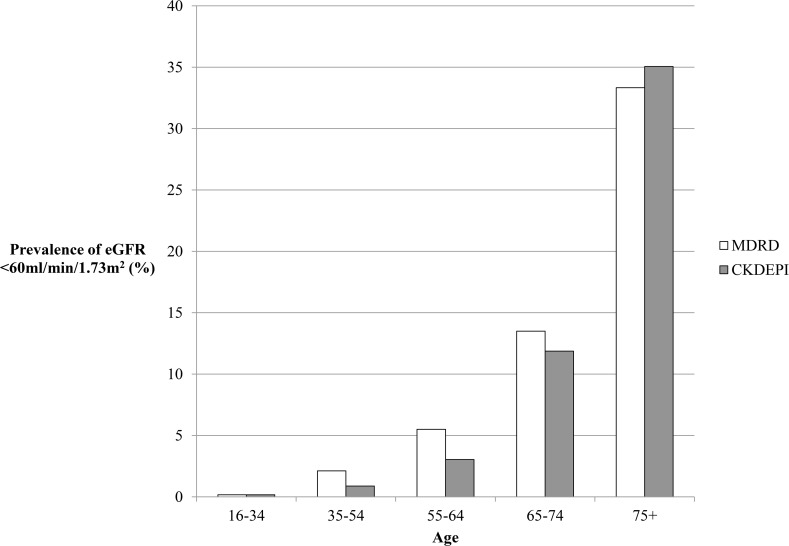
Prevalence of eGFR<60ml/min/1.73m^2^ by age grouping and serum creatinine eGFR estimating method.

Use of the CKDEPI Scr equation classified fewer individuals as having eGFR<60ml/min/1.73m^2^ than the MDRD equation (5.2% vs. 6.0%, Table 2). About 1% of all individuals would be classified as having CKD G3-5 by the MDRD equation that would not by CKDEPI, compared with only 0.2% for CKDEPI equation. Estimated prevalence was lower in both sexes using the CKDEPI Scr equation but this varied by age, with a higher estimated prevalence in people over 75 (Fig. 1). People reclassified by CKDEPI as not having eGFR<60ml/min/1.73m^2^ were more likely to be younger, female, and less likely to have factors associated with poorer outcome such as diabetes, hypertension and albuminuria. Based on 2011 Census data, the estimated number of adults aged 16 and above with eGFR<60ml/min/1.73m^2^ in England was 2.59 million (MDRD) and 2.25 million (CKDEPI). A change from routine use of MDRD to CKDEPI would therefore result in over 340,000 fewer adults having eGFR<60ml/min/1.73m^2^ in England.

**Table 2:** Sociodemographic and clinical characteristics of people with CKD 3–5 defined by eGFR (from MDRD and CKDEPI equations) and after targeted addition of cystatin C.

		MDRD eGFR <60ml/min/1.73m^2^		CKDEPI eGFR <60ml/min/1.73m^2^		At least two biomarkers (CKDEPI eGFR <60ml/min/1.73m^2^, uACR> = 3mg/mmol, cystatin C eGFR <60ml/min/1.73m^2^)	
**Total in category**		349 / 5786		303 / 5786		230 / 5786	
% of study total (95% confidence intervals)		6 (5.4–6.6)		5.2 (4.7–5.8)		4.0 (3.5–4.5)	
		n	column %	n	column %	n	column %
**Sex**	**Male**	135	38.7	126	41.6	115	50.0
	**Female**	214	61.3	177	58.4	115	50.0
**Age**	**16–54**	46	13.1	21	6.9	10	4.3
	**55+**	303	86.9	282	93.1	220	95.7
**Diabetes**	**(total)**	70	20.1	66	21.9	59	25.7
**Hypertension**	**(total)**	241	61.9	224	74.2	183	79.6
**Albuminuria**	**A1**	246	79.1	207	75.9	128	55.7
	**A2 and A3**	65	20.9	66	24.1	102	44.3

eGFR: Estimated glomerular filtration rate

MDRD: Modification of diet in renal disease

CKDEPI: Chronic Kidney Disease Epidemiology Collaboration

uACR: Urinary albumin to creatinine ratio.

**Fig 2:**
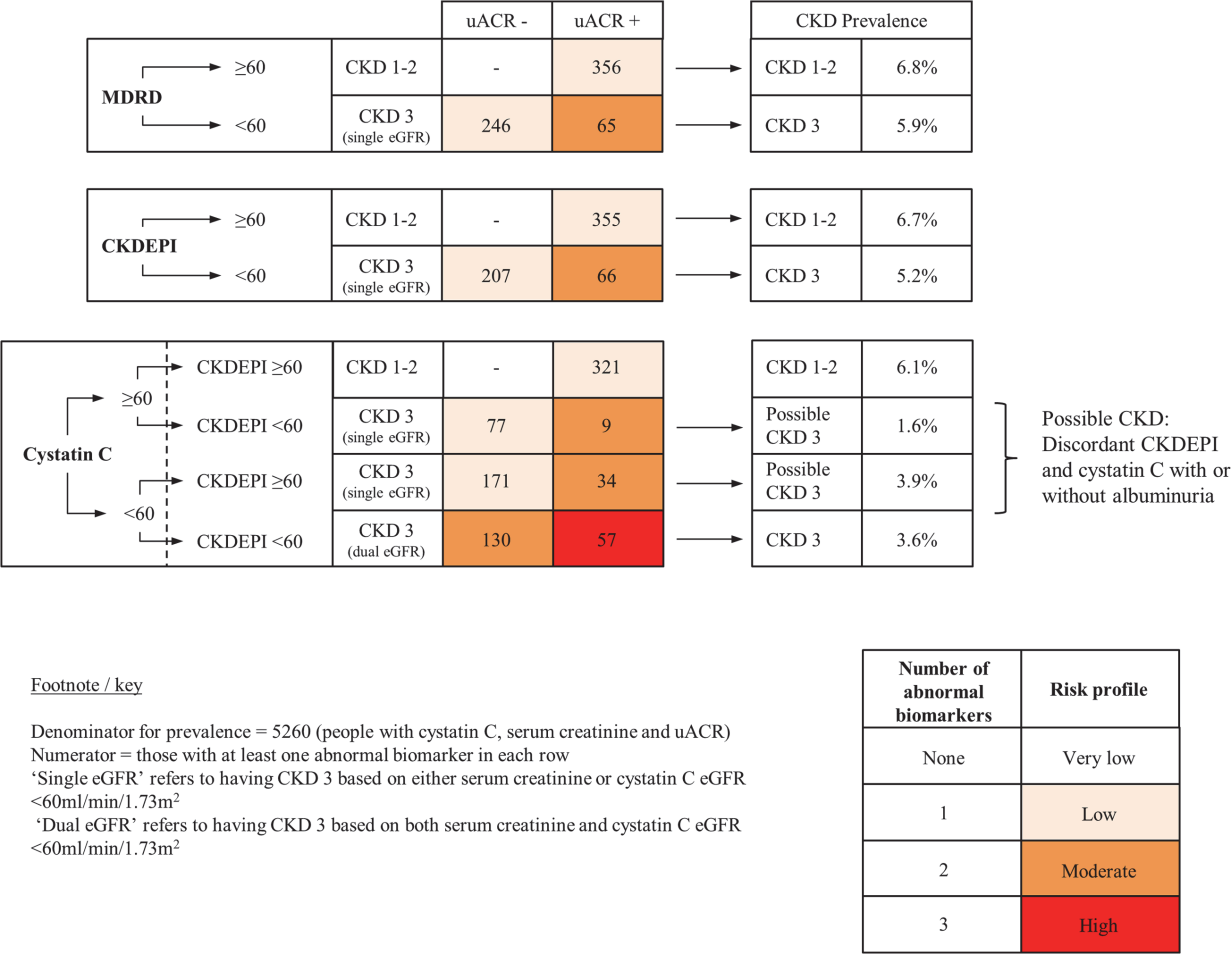
Effect of the use of different combinations of measures on the risk profile and estimated prevalence of CKD.

The prevalence of cystatin C-derived eGFR<60ml/min/1.73m^2^ was 447/5786 (7.7%), higher than both CKDEPI and MDRD equations. Measurement of uACR and cystatin C in addition to CKDEPI eGFR reduced those classified as having potential moderate CKD (KDIGO categories G3a and 3b) to 230 (estimated prevalence 4%, Table 2).[3] Fig. 2 summarises the effect of the use of the different measures on the risk profile and estimated prevalence of CKD among those tested for all three biomarkers (n = 5260). In total, 799/5260 (15.2%) had at least one abnormal biomarker. Applying cystatin C to people with CKDEPI-defined eGFR <60ml/min/1.73m^2^ (representing possible CKD category G 3–5) and people with CKDEPI-defined eGFR ≥60ml/min/1.73m^2^ (representing CKD category G 1–2) resulted in the following CKD groups:

In the 207 (3.9%) people with CKDEPI-defined eGFR<60ml/min/1.73m^2^ without albuminuria (CKD Category G3a A1) about a third (77, 1.4% of total) were reclassified into a lower risk group with one of three biomarkers and two thirds (130, 2.5% of total) into a group with two of three. Among people aged over 75, there were 91 with CKDEPI-defined eGFR<60ml/min/1.73m^2^ without albuminuria (CKD Category G3a A1). Of those, 22 (24.2%) were reclassified as having no CKD by the addition of cystatin C eGFR.

In the 355 (6.7%) people with CKDEPI eGFR ≥60ml/min/1.73m^2^ with albuminuria (CKD Category G1-2) almost a tenth (34, 0.6% of total) were reclassified into a higher risk group with two positive biomarkers.

In the 66 (1.3%) people with CKDEPI-defined eGFR <60ml/min/1.73m^2^ and albuminuria (CKD Category G3-5 A2-3) over 85% (57,1.1% of total) had all three biomarkers when cystatin C measurement was added.

Table 3 compares some key characteristics of individuals with albuminuria only and CKDEPI eGFR <60ml/min/1.73m^2^ only with those who have combinations of abnormal markers. Although the numbers are small, this suggests that those with albuminuria and cystatin C abnormalities tend to be older, male and a higher proportion have hypertension than those with albuminuria alone. It also suggests that those with creatinine as well as cystatin C abnormality tend to be older and have a higher proportion with diabetes and hypertension than those with creatinine <60ml/min/m^2^ alone.

**Table 3:** Characteristics of populations with selected combinations of biomarkers following testing with cystatin C.

Number of abnormal biomarkers		1				2						3	
Biomarker combinations		Albuminuria alone		Creatinine <60 ml/min/1.73m^2^ alone		Albuminuria & Cystatin C <60 ml/min/1.73m^2^		Creatinine & Cystatin C <60 ml/min/1.73m^2^		Albuminuria & Creatinine <60 ml/min/1.73m^2^		Albuminuria + Cystatin C & Creatinine <60 ml/min/1.73m^2^	
**Total in category**		321 / 5260		77 / 5260		34 / 5260		130 / 5260		66 / 5260		57 / 5260	
% of total (95% confidence intervals)		6.1 (5.5–6.8)		1.5 (1.1–1.8)		0.6 (0.4–0.9)		2.5 (2.1–2.9)		1.3 (1.0–1.6)		1.1 (0.8–1.4)	
		n	%	n	%	n	%	n	%	n	%	n	%
**Sex**	**Male**	161	50.2	24	31.2	23	67.6	57	43.8	36	54.5	30	52.6
	**Female**	160	49.8	53	68.8	11	32.4	73	56.2	30	45.5	27	47.4
**Age**	**16–54**	192	59.8	13	16.9	4	11.8	3	2.3	1	2.2	4	8.0
	**55+**	129	40.2	64	83.1	30	88.2	127	97.7	65	97.8	53	92.0
**Diabetes**	**(total)**	49	15.3	5	6.5	3	8.8	33	25.4	22	33.3	19	33.2
**Hypertension**	**(total)**	191	59.5	54	70.1	28	82.4	117	90.0	56	85.8	48	84.8
**BMI**	**<25**	97	32.4	15	22.1	6	24.0	10	7.7	12	21.7	10	21.7
	**25 to <30**	117	39.1	28	41.2	10	40.0	44	33.8	24	45.2	21	45.7
	**≥30**	85	28.5	25	36.7	9	36.0	47	36.2	18	33.1	15	32.6

BMI: Body Mass Index

### Distributions and associations of cystatin C

The mean cystatin C level was 0.96mg/L (SD 0.27) and median 0.92mg/L (IQR 0.82–1.03) Distribution of cystatin C varied by age and sex. At younger ages, males had higher median cystatin C than females, but this gender difference narrowed as age increased. In univariate analyses, increasing age, lack of qualifications, smoking, increasing BMI, diabetes, hypertension, total cholesterol, HDL cholesterol were all significantly positively associated with cystatin C eGFR<60ml/min/1.73m^2^. In the fully adjusted model, increasing age, lack of qualifications, being an ex-smoker, increasing BMI, diabetes, hypertension, and albuminuria remained associated (Table 4). There were similar associations for elevated cystatin C using the derived threshold 1.2mg/l. There were no significant interactions.

**Table 4:** Estimated prevalence and associations of cystatin C CKD 3–5 (Grubb-defined eGFR <60ml/min/1.73m^2^) with socio-economic and clinical factors.

Variable		Estimated prevalence of cystatin C (Grubb) eGFR <60ml/min/1.73m^2^	Associations of cystatin C (Grubb) eGFR <60ml/min/1.73m^2^		
			Univariate	Age sex adjusted	Multivariable^†^
		Row %	OR (95%CI)	OR (95%CI)	OR (95%CI)
**Sex**	Male	7.5	1	1	1
	Female	7.9	1.06 (0.87–1.29)	0.87 (0.69–1.08)	0.89 (0.66–1.20)
**Age**	16–34	0.7	1	1	1
	34–54	1.9	3.62 (1.82–7.21)**	3.61 (1.81–7.20) **	3.02 (1.41–6.45) **
	55–64	6.3	12.44 (6.35–24.38)**	12.46 (6.36–24.43) **	7.53 (3.50–16.22) **
	65–74	15.9	33.63 (17.50–64.64)**	33.72 (17.54–64.82) **	19.19 (9.06–40.68) **
	75+	44.3	140.37 (79.94–266.48)**	142.24 (74.89–270.17) **	77.38 (36.44–164.29) **
**Qualification**	Degree	2.1	1	1	1
	Below degree	5.3	2.61 (1.74–3.93) **	2.16 (1.40–3.33) **	1.91 (1.16–3.14)*
	None	20.3	11.84 (7.90–17.75) **	3.35 (2.16–5.20) **	2.67 (1.60–4.45) **
**Smoker**	Never	5.8	1	1	1
	Ex	12.9	2.40 (1.94–2.98) **	2.42 (1.75–3.34) **	2.21 (1.51–3.23) **
	Current	6.4	1.11 (0.85–1.47) **	1.41 (1.1–1.81) **	1.23 (0.91–1.67)
**BMI**	Normal	3.8	1	1	1
	Overweight	6.9	1.86 (1.39–2.48) **	1.32 (0.96–1.83)	1.35 (0.94–1.95)
	Obese	9.9	2.77 (2.06–3.71) **	2.16 (1.55–3.00) **	1.93 (1.30–2.85) **
**Diabetes (doctor diagnosed)**	No	6.7	1	1	1
	Yes	24.3	4.08 (3.07–5.42) **	2.19(1.58–3.05) **	1.73 (1.15–2.60) **
**Hypertension (doctor diagnosed)**	No	4.3	1	1	1
	Yes	18.0	4.91 (4.02–5.99) **	1.74 (1.39–2.19) **	1.45 (1.10–1.93)*
**Albuminuria**	None	6.2	1	1	1
	Micro	20.9	3.99 (3.05–5.21) **	2.29 (1.66–3.15) **	1.96 (1.36–2.82) **
	Macro	31.8	6.83 (2.74–16.99) **	7.19 (2.36–21.90) **	6.97 (2.22–21.93) **
**Total cholesterol (mmol/L)**	Continuous	8.5	1	1	1
		7.0	1.24 (1.02–1.51) **	1.11 (1.01–1.22) *	1.01 (0.89–1.13)
**HDL cholesterol (mmol/L)**	Continuous	9.5	1	1	1
		7.2	1.35 (1.09–1.68) **	1.89 (1.42–2.52) **	1.32 (0.92–1.88)

† Adjusted for age, sex, qualification, smoking, BMI, Doctor-diagnosed-hypertension and diabetes, albuminuria, total cholesterol, HDL cholesterol and HBA_1c_.

* p<0.05

**p<0.01

BMI: Body Mass Index

HDL: High Density Lipoprotein

OR: Odds ratio

eGFR: Estimated glomerular filtration rate.

## Discussion

This study provides the first population-level comparison of low eGFR defined by serum creatinine-based (MDRD and CKDEPI) equations and by cystatin C in England; the first descriptions of the distribution and associations of serum cystatin C concentration; and the potential effects of its targeted use to improve risk stratification.

It suggests that the prevalence of eGFR<60ml/min/1.73m^2^ in England would be lower by 0.8% if the CKDEPI equation was introduced to classify CKD, equivalent to about 340,000 fewer people. Estimated CKD prevalence would be reduced in all age groups except in people over 75, and those still classified as CKD would be at higher risk of adverse consequences, as others have found.[13,20,22,24] This study also adds that targeted use of cystatin C in people with one or two abnormal biomarkers (Scr-based eGFR<60ml/min/1.73m^2^ and / or uACR≥3mg/mmol, about 12% of the population in this study) would further reduce the estimated prevalence of people labelled as having CKD and likely improve their risk stratification.

Several other studies have reported a lower prevalence of CKD G3–5 with the use of the CKDEPI equation. In the Australian Diabetes, Obesity and Lifestyle Study, CKDEPI reclassified approximately 1.9% of individuals as not having CKD G3–5 compared with MDRD.[32] In the study by O’Callaghan and colleagues in the UK, the change of equation from MDRD to CKDEPI resulted in a reduction in the number of people with eGFR<60ml/min/1.73m^2^ from 27,579 to 25,504 out of 321,964 tests (a relative fall of 7.5%), resulting in a reduction in prevalence of approximately 1.2% of all patients tested. Our study suggests a more modest reduction in prevalence, probably because it was representative of the population as a whole, rather than being conducted on routine specimens as in the O’Callaghan study [13] and thus included a higher proportion of individuals with no CKD. On the other hand, Cystatin C-based methods alone would classify a higher proportion of the population as having eGFR<60ml/min/1.73m^2^, and therefore CKD G3–5, compared with Scr, both using the Grubb equation to derive eGFR, or using cystatin C directly with a threshold level, especially in older people. This is consistent with data from NHANES in the US showing that the prevalence of elevated cystatin C levels increased dramatically with age, with over 50% of those older than 80 having elevated levels.[21] Analyses of CKD prevalence trends in NHANES suggest higher prevalence of CKD G3–5 derived from cystatin C than from creatinine based equations.[33]

We identified significant associations between elevated cystatin C and increasing age, lower qualification, smoking, higher BMI, diabetes, hypertension, raised total cholesterol, lower HDL cholesterol, and albuminuria after adjusting for age and sex, although not all of these associations remained on full adjustment. In our study, the associations of elevated cystatin C and Grubb-defined eGFR<60ml/min/1.73m^2^ were very similar. Similar associations with elevated cystatin C (using a threshold method) were identified in NHANES (older age, hypertension, current smoking, higher BMI, and higher triglyceride levels).[21] Cystatin C has been associated with overweight and obesity in the general population,[34] and with the metabolic syndrome in people with dyslipidaemias independent of serum creatinine.[35] Several studies have demonstrated that cystatin C identifies people at higher risk of CKD progression and complications more strongly than Scr eGFR.[20,24,36] Despite these advantages, the higher prevalence of people labelled as having CKD, the higher cost of cystatin C assay and the lack of specificity for CKD are likely to make the use of cystatin C as the first line measure of kidney function (or as a screening tool for CKD) impractical, but it might have an important role in risk stratification in those with CKD defined by Scr eGFR and uACR, as recommended in the revised NICE guidelines.[12]

Non GFR determinants of cystatin C have been recognised as a limitation for its individual prognostic value.[37] However, several studies in a variety of populations have shown the value of adding cystatin C to existing markers as part of assessing risk in people with CKD.[20,36,38] In the Reasons for Geographic and Racial Differences in Stroke (REGARDS) study, a prospective cohort of over 26,000 adults, Peralta et al demonstrated the value of a triple marker approach using creatinine, uACR and cystatin C to identify people at higher risk of both mortality and CKD progression.[24] Abnormality of all three biomarkers was associated with the highest risk of all-cause mortality (HR 5.6 (95% confidence interval 3.9–8.2)) in the Peralta study, and risks were similar for those with CKD defined by creatinine and uACR(HR 3.3 (95% confidence interval 2.0–5.6)) and those defined by creatinine and cystatin C (HR 3.2 (95% confidence interval 2.2–4.7)). A similar pattern was seen for incident ESKD.[24] In a meta-analysis of 11 general population studies with over 90,000 participants, the CKD Prognosis Consortium has demonstrated that use of cystatin C in combination with creatinine strengthens the association between eGFR and risk of death or end stage kidney disease.[39] Revised UK NICE CKD guidelines propose the use of cystatin C in those with Scr eGFR 45–59ml/min/1.73m^2^ and uACR<3mg/mmol if confirmation of CKD is required.[12] Although we were unable to demonstrate the link with clinical outcomes because of the cross sectional nature of the HSE, our study suggests that a significant proportion (about a third) of people in England currently defined as having CKD category G3a by creatinine based eGFR alone (CKDEPI) could be stratified as being at lower risk by such targeted use of cystatin C testing. This would help allay some of the current concerns of over diagnosis and inappropriate disease labelling.[8] We also identified a small sub group at increased risk from the larger group with CKD G1–2. Such risk stratification would provide information for better targeting of interventions such as improved blood pressure control, use of renin angiotensin aldosterone system inhibitors, cardiovascular risk reduction and acute kidney injury (AKI) avoidance. Addition of cystatin C might also improve current risk stratification models that have greater utility in predicting CKD progression than cardiovascular risk.[40] The cost of cystatin C testing in routine practice has not been assessed in this study but the revised NICE CKD guidance included economic analysis.[41] This estimated that, while increased cystatin C testing increases the costs of CKD diagnosis, the fall in the number of people requiring treatment and monitoring is likely to result in overall cost saving.[41] Patients with an eGFR of <60 ml/min/1.73m^2^ are currently listed on primary care kidney disease registers which form part of the incentive payment Quality and Outcomes Framework (QOF) funding arrangement in England.[42] Reduced prevalence of CKD G3–5 could therefore result in reduced incentive payments to General Practitioners. This could be addressed by a higher payment for the smaller number assessed more accurately.

### Strengths and limitations

The HSE 2009 and 2010 are nationally representative samples, pooled over two years to increase numbers and precision of estimates. There were standardised protocols for measurement by trained interviewers and nurses. All samples were tested in the same laboratory with standardised assays, which overcomes issues of selective testing in routine practice. There was weighting for non-response to reduce response bias.

However the study was limited by its cross-sectional nature, which restricts the ability to infer causal relationships from the associations identified. While we have shown that cystatin C is associated with important risk factors (and thus may improve risk stratification), we could not analyse its independent predictive capacity and can therefore not be certain that it would improve CKD risk stratification. However, other studies such as that by Peralta et al strongly suggest that it would.[24] Although no ‘gold standard’ measure of GFR was available to assess ‘true’ prevalence, that will always be the case for large, general population surveys and indeed is what occurs in routine practice. In addition, only a single blood sample was taken for measurement of serum cystatin C and creatinine, and a single sample of urine tested for uACR. Fluctuations in creatinine have been shown to have a strong influence on CKD prevalence.[43]

Generalizability is also limited by the assay used to determine cystatin C value (the particle-enhanced turbidimetric assay (PETIA)) and the manufacturer’s recommended equation to derive eGFR (Grubb), in contrast to the majority of previous studies that have used the particle-enhanced nephelometric assay (PENIA). Biases have been demonstrated between these assays; PETIA cystatin C values were 27.5% higher than PENIA results and PENIA results were 12.9% lower in 2010 than in 2000.[44] Such findings have led to calls for standardisation of cystatin C assays.[45] Recent development of a CKDEPI creatinine-cystatin C equation (an eGFR equation combining cystatin C with standardised serum creatinine) using PENIA has demonstrated improved diagnostic accuracy of the combined equation compared with either marker alone for measured GFR.[23] We were unable to use the CKDEPI-cystatin C eGFR equation or this combined equation due to the PETIA cystatin C assay method used, though the different methods have shown similar characteristics in predicting cardiovascular outcomes.[46]

Prevalence of category G4 and 5 CKD is likely to be underestimated as, while the HSE is able to adjust for non-response among the general population in private households, it may not fully account for some in whom more severe CKD (category G4 and 5) will be more common. This would include those who were not able to give a blood or urine sample because of poor health and those who did not participate due to concurrent illness or hospitalisation, as well as those who were in residential care.

## Conclusion

In this nationally representative population based study, estimated prevalence of CKD was lower using the CKDEPI equation to classify CKD rather than the currently used MDRD equation, and those identified were likely to be at higher risk of complications and kidney disease progression. Selected use of cystatin C may further improve risk stratification, and address some of the current concerns about over diagnosis and over treatment of CKD G3.
